# Panel testing for the molecular genetic diagnosis of congenital hypogonadotropic hypogonadism – a clinical perspective

**DOI:** 10.1038/s41431-022-01261-0

**Published:** 2022-12-15

**Authors:** Yasmin Al Sayed, Sasha R. Howard

**Affiliations:** 1 Centre for Endocrinology, William Harvey Research Institute, Queen Mary University of London, London, UK; 2 Department of Paediatric Endocrinology, Royal London Children’s Hospital, Barts Health NHS Trust, London, UK

**Keywords:** Hypogonadism, Genetic testing

## Abstract

Congenital hypogonadotropic hypogonadism (CHH) is a rare endocrine disorder that results in reproductive hormone deficiency and reduced potential for fertility in adult life. Discoveries of the genetic aetiology of CHH have advanced dramatically in the past 30 years, with currently over 40 genes recognised to cause or contribute to the development of this condition. The genetic complexity of CHH is further increased by the observation of di- and oligogenic, as well as classic monogenic, inheritance and incomplete penetrance. Very recently in the UK, a panel of 14 genes has been curated for the genetic diagnosis of CHH within the NHS Genomic Medicine Service programme. The aim of this review is to appraise the advantages and potential pitfalls of the use of a CHH panel in clinical endocrine diagnostics, and to consider the future avenues for developing this panel including the potential of whole exome or whole genome sequencing data analysis in this condition.

## Introduction

### Congenital hypogonadotropic hypogonadism

**Fig. 1:**
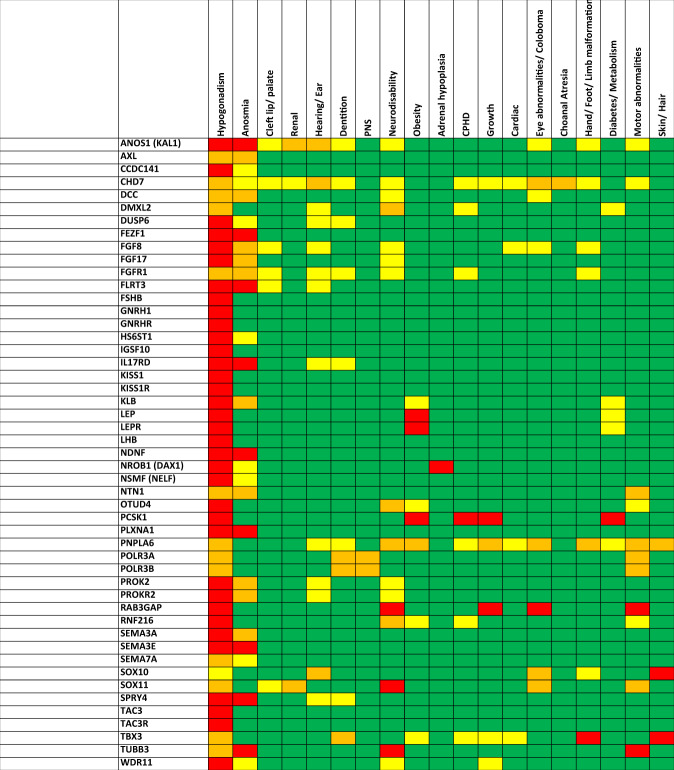
‘Heat map’ showing frequency of phenotype–genotype associations with gene mutations most often identified in individuals presenting with congenital hypogonadotropic hypogonadism (CHH) or Kallmann Syndrome (KS). Colour code for organ involvement: Red, typically present; orange, mostly present; yellow, sometimes seen; green, not described. Previous gene name given in brackets.

Congenital hypogonadotropic hypogonadism (CHH, MIM 146110, 614837, 615266, 615267, 615269, 615270, 615271, 614880) is a rare disease that results in a lack of normal pubertal development and reproductive immaturity. The condition is characterised by low circulating sex steroid concentrations resulting from a deficiency of pituitary gonadotropin production. The central defect is usually in the development of the gonadotropin-releasing hormone (GnRH) neuronal network in the hypothalamus in foetal life, but may also be secondary to defects in downstream pituitary gonadotrope pathways [1]. It has an estimated incidence of 1:15,000–50,000, with a male to female predominance of 3.6:1 [2, 3]. CHH often presents with delayed or absent puberty in adolescence, but may also be indicated by the presence of ‘red flag’ features such as anosmia (when the condition is termed Kallmann Syndrome, KS, MIM 308700, 147950, 244200, 610628, 612370, 612702), cryptorchidism or micropenis (which may allow diagnosis of males in infancy), synkinesis (mirror movements) and midline defects such as cleft palate or renal agenesis [4]. CHH may additionally be part of a wider genetic syndrome involving other pituitary hormone deficiencies (combined pituitary hormone deficiency, CPHD), neurodevelopmental disorders (for example with Coffin-Siris syndrome [5] or PEPNS [6]), and other non-reproductive phenotypes [7] (Fig. 1).

### Clinical diagnosis and management of CHH

Unlike with many other rare diseases, CHH is amenable to treatment with replacement of either GnRH, gonadotropin or sex steroid hormones [8]. Whilst early intervention is optimal, even with treatment in adult life interventions can result in restoration of fertility in both men and women in approximately 75% of patients [9, 10]. However, both delayed presentation and diagnosis are common in this condition, as clinical assessment can be challenging with subtle symptoms and signs of hypogonadism that can be missed [11]. There are many instances of first diagnosis in adult life, when patients present with hypogonadism, lack of libido and reduced energy levels and bone density, or are diagnosed during investigations for infertility [4]. In women, where the condition is less prevalent it is also less frequently considered, and, without the red flag features of micropenis, cryptorchidism and small volume testes that may be diagnostic clues in men, can present an even more elusive clinical picture.

This diagnostic challenge is particularly pertinent for paediatric endocrinologists looking after adolescents with delayed puberty [12]. In these patients, the presentation of isolated or constitutional delay in puberty – a common and usually self-limiting condition - may be indistinguishable from CHH by traditional biochemical investigations [13]. Both conditions will present with low sex steroid and gonadotropin concentrations, and even recently adopted biomarkers (such as inhibin B) do not have sufficient sensitivity and specificity to discriminate GnRH deficiency from constitutional delay [12, 14]. Additionally, patients with partial CHH may enter puberty but then stall, lending further complexity to the diagnostic algorithm.

Therefore, the potential for genetic testing to investigate a patient with suspected CHH or KS, in order to make a clear diagnosis where clinical phenotypic and biochemical features have not provided one, is an attractive avenue to pursue. The imperative to make this diagnosis in a timely manner in adolescence is driven not only by the need to reduce invasive and costly investigations and uncertainty for the patient, but also because the timing and nature of treatment differs depending on the underlying condition [15]. For patients with CHH, therapy to induce puberty will be given promptly and at an earlier age and male patients can be offered treatment with gonadotropins to increase their potential for future fertility [16]. Delays in diagnosis have also been shown to lead to significant psychological and emotional distress for the patients and their families [17].

## Genetic diagnosis in CHH

### The genetic aetiology of CHH

As with many rare diseases, the era of high throughput sequencing has facilitated our understanding of the genetic aetiology of CHH, with the identification of more than 40 genes that are implicated in its development (Table 1) [18]. For patients with CPHD or septo-optic dysplasia there are many additional genes that have been identified to contribute to the aetiology, which have been reviewed extensively in the literature and are beyond the scope of this review [19]. In addition to monogenic inheritance, di- and oligogenic mutational burden as well as incomplete penetrance has been observed in CHH pedigrees [20–22]. Despite the clear genetic heterogeneity, recent studies using molecular genetic diagnosis in large cohorts with CHH or KS show that an underlying variant of interest can be identified in 21–51% of patients [18, 21, 23, 24]. Many of the genes in which loss-of-function variants are identified in individuals with CHH encode receptor-ligand pairs, for example: *GNRH1* (MIM 152760) and its receptor (encoded by *GNRHR*, MIM 138850); and *Kisspeptin-1* (*KISS1*, MIM 603286) and its receptor *KISS1R* (MIM 604161). Autosomal dominant, autosomal recessive and X-linked inheritance have been identified, as well as rare cases of uniparental isodisomy [25]. Evidence of phenotype-genotype correlation can aid targeted genetic testing, for example if syndromic features are present such as hearing loss, severe obesity or skeletal abnormalities (Fig. 1).

**Table 1:** Genes of interest identified in cohorts of patients with congenital hypogonadotropic hypogonadism (CHH) or Kallmann Syndrome (KS), grouped by frequency (identified in >1% or <1% of patients in CHH/KS patient cohorts) and for known syndromes with associated CHH/KS.

Gene	OMIM	Cytogenetic location
*Frequently Identified in Cohorts of CHH/KS*		
*FGFR1*	147950	8p11.23
*ANOS1 (KAL1)*	300836	Xp22.31
*CHD7*	612370	8q12.2
*PROKR2*	244200	20p12.3
*GNRHR*	146110	4q13.2
*KISS1R*	614837	19p13.3
*TAC3*	614839	12q13.3
*TACR3*	614840	4q24
*FGF8*	612702	10q24.32
*FGF17*	603725	8p21.3
*PROK2*	610628	3p13
*CCDC141*	616031	2q31.2
*SEMA3A*	614897	7q21.11
*IL17RD*	615267	3p14.3
*AXL*	109135	19q13.2
*HS6ST1*	614880	2q14.3
*Less Frequently Identified in Cohorts of CHH/ KS*		
*GNRH1*	614841	8p21.2
*KISS1*	614842	1q32.1
*NDNF*	616506	4q27
*DCC*	120470	18q21.2
*NTN1*	601614	17p13.1
*KLB*	611135	4p14
*DUSP6*	602748	12q21.33
*SPRY4*	607984	5q31.3
*IGSF10*	617351	3q25.1
*FSHB*	136530	11p14.1
*LHB*	152780	19q13.33
*NSMF (NELF)*	614838	9q34.3
*WDR11*	614858	10q26.12
*SEMA7A*	607961	15q24.1
*SEMA3E*	608166	7q21.11
*PLXNA1*	601055	3q21.3
*FEZF1*	613301	7q31.32
*FLRT3*	604808	20p12.1
*TUBB3*	602661	16q24.3
*NROB1 (DAX1)*	300200	Xp21.2
*LEP*	614962	7q32.1
*LEPR*	614963	1p31.3
*PCSK1*	162150	5q15
*Syndromes associated with CHH/ KS*		
*CHD7 –* CHARGE syndrome	612370	8q12.2
*DMXL2 -* Polyendocrine-Polyneuropathy Syndrome	612186	15q21.2
*OTUD4 -* Gordon Holmes syndrome	611744	4q31.21
*RNF216 -* Gordon Holmes syndrome	212840	7p22.1
*PNPLA6 -* Oliver-McFarlane/ Laurence-Moon Syndrome	603197	19p13.2
*POLR3A -* Hypomyelinating leukodystrophy-8 with hypogonadotropic hypogonadism (4H syndrome)	614258	10q22.3
*POLR3B -* Hypomyelinating leukodystrophy-8 with hypogonadotropic hypogonadism (4H syndrome)	614366	12q23.3
*RAB3GAP1 –* Warburg Micro syndromeand	602536	2q21.3
*RNF216 -* Gordon Holmes syndrome	212840	7p22.1
*SOX10 -* Waardenburg syndrome	602229	22q13.1
*SOX11 –* Coffin Siris Syndrome	600898	2p.25.2
*TBX3 –* Ulnar-Mammary syndrome	601621	12q24.22

Previous gene names are given in brackets (Data sources [4, 21, 23, 24, 44].

Whilst there is some overlap between the genetic pathways contributing to the conditions of CHH, isolated (self-limited) delayed puberty and the adult-onset hypogonadism condition termed hypothalamic amenorrhoea [26], there are clear distinctions in their mutational signatures [13, 24]. Understanding of the genetic aetiology of isolated delayed puberty has also progressed rapidly over the last decade and may thus help to distinguish the diagnosis of this condition from CHH in adolescence [27]. The use of molecular genetic diagnosis is thus vitally important to understand the pathophysiological basis of the disease, in order to optimise and personalise patient management [15].

### Molecular genetic diagnosis via PanelApp

In view of the above background, the opportunity to carry out molecular genetic testing for CHH in a timely manner is welcomed by endocrine clinicians. In the UK, this has very recently become available for patients in paediatric and adult endocrine services through the National Health Service (England) (NHSE) Genomic Medicine Service (GMS), which has been set up to deliver genomic testing through a network of genomic laboratory hubs [28]. Incorporated within this service is the Genomics England PanelApp programme, an open-access platform developed to support virtual gene panel curation by genomic experts [29]. Guidance on panel design and maintenance has been provided by the American College of Medical Genetics and Genomics (ACMG), including on incomplete penetrance and technical considerations [30]. Gene panel versions used for diagnosis in the NHSE are overseen by the NHSE Genomics Clinical Reference Group and reviewed by test evaluation working groups. PanelApp has also embedded a review tool to allow each gene to be reviewed and commented on by experts in the scientific community [29]. To date, 173 panels have been developed within the PanelApp programme for diagnostic use. These gene panels use a traffic light system that is dependent on the current evidence of a particular gene’s involvement in the disease [29] (Table 2). The “green” rating indicates genes above a certain evidence threshold that can be used in diagnostic reporting. Guidelines for the level of evidence required for a gene to be classified as “green” were based on existing ClinGen [31] and Deciphering Developmental Disorders (https://www.deciphergenomics.org/ddd/overview) [32] project gene guidelines. These “green” genes require case-level evidence from three unrelated families or two unrelated families with convincing functional data. In addition, there must be evidence that “disease-causing mutations follow a Mendelian pattern of causation appropriate for reporting in a diagnostic setting” and there is no convincing contradictory evidence [29]. “Amber” and “red” ratings reflect gene-disease associations with moderate or low levels of evidence, respectively, which will not be included in the diagnostic report, but are maintained as lists of genes that may be useful for research purposes or may be promoted to green with future supporting evidence emerges. The current pipeline is that information about variants of interest in genes that are rated “green” will be fed back to the patients’ clinicians, but variants of interest in “amber” or “red” genes will not.

**Table 2:** NHS Genomic Medicine Panel App R148, Hypogonadotropic hypogonadism idiopathic genes, version v1.4 (https://nhsgms-panelapp.genomicsengland.co.uk/panels/650/v1.4).

	

Genes are coloured via a traffic light system (green, yellow, red) based on evidence for relevance for the condition, with variants of interest in ‘green’ genes being reported to clinicians. Gene in grey (NDNF) is still under review.

### PanelApp hypogonadotropic hypogonadism panel (R148)

The R148 Hypogonadotropic Hypogonadism panel (https://nhsgms-panelapp.genomicsengland.co.uk/panels/650/v1.4) includes 14 genes which are rated “green”, with an additional 6 genes rated “amber” and 12 genes that are graded “red” (Table 2). Of note, there is a separate panel for patients with CPHD. These 14 genes that are clinically reportable are: *ANOS1*, *CHD7*, *FGF8*, *FGFR1*, *FSHB*, *GNRHR*, *IL17RD*, *KISS1R*, *LHB*, *PROK2*, *PROKR2*, *TAC3*, *TACR3*, *WDR11*. Of these, most fall into the category of genes in which pathogenic variants are frequently (>1% of patients) identified in cohorts of non-syndromic HH or KS (Table 1). Thus, genes such as *ANOS1* (MIM 300836) [33, 34], *CHD7* (MIM 608892) [7, 35], *FGFR1* (MIM 136350) [36, 37], *PROKR2* (MIM 607123) [38, 39], *GNRHR* (MIM 138850) [38, 40], *TACR3* (MIM 162332) [41, 42] – indeed the majority of these 14 genes – have been repeatedly validated in international cohorts of patients with CHH and KS, with associated in vitro and animal model functional evidence of the molecular mechanisms by which they contribute to the disease and pathogenicity of disease variants [18, 21, 23, 43, 44]. Whilst luteinizing hormone beta (*LHB*) and follicle-stimulating hormone beta (*FSHB*) are not frequently identified with pathogenic variants in CHH cohorts, they have clear biological basis and a strong evidence base for inclusion [45–48]. Loss-of-function variants in *FSHB* and *LHB* have been associated with infertility, primary amenorrhoea, azoospermia and variable impairment of pubertal development [48–51].

In contrast, variants of interest in *WDR11* are more rarely identified in cohorts with CHH; and have less evidence for their candidacy as key genes for the pathogenesis of this disease. *WDR11* (WD repeat domain 11, MIM 606417) is a gene that has been previously found to be involved in tumorigenesis of human glioblastoma cells [52]. Five different missense heterozygous variants in the *WDR11* gene (NM_172255.3: c.1343G>A, p.R448Q; c.1303G>A, p.A435T; c.2070T>A, p.H690Q; c.1183C>T, p.R395W; c.3450T>G, p.F1150L) were first identified in seven patients from a cohort of CHH families in 2010 [53]. Its WD domain was reported to interact with the homeodomain transcription factor EMX1, which is implicated in the development of olfactory neurons [54]. Interaction of WDR11 variants with EMX1 was either abolished or reduced in all but one identified variant (NM_018117: c.1183C>T, p.R395W) [53]. The same group also identified in 2018 an additional patient with CHH with a novel heterozygous variant (NM_018117: c.1610C>T, p. P537L), inherited from their mother who had a normal phenotype, thus suggesting an incomplete penetrance of the variant [55]. Two very recent CHH cohort studies used high throughput sequencing to identify a novel probably pathogenic variant (NM_018117.12: c.731T>C, p.L244P) [56] and a predicted truncating variant (NM_018117.12: c.163dup, p.G55P fs7*) [57] in this gene in patients with KS. Further pleiotropy is evidenced from screening of 37 families with pituitary stalk interruption syndrome using whole exome sequencing, which identified three patients with CHH who harboured two different predicted pathogenic missense variants in *WDR11* (NM_018117: c.T109G, p.Y37D and c.G3571A, p.G1191S) and one with a predicted essential splice site loss of function variant (NM_018117: c.199–9T>C) [58]. Moreover, another recent publication demonstrated that biallelic loss-of-function variants in *WDR11* result in a more pronounced phenotype of short stature, pronounced microcephaly and intellectual disability as observed in six patients from three independent families [59]. These features are in keeping with the mouse *Wdr11*-knockout model which has features of holoprosencephaly, cardiac defects, pituitary dysgenesis and hypogonadism [55]. None of the probands’ parents, who were heterozygous for *WDR11* variants showed any phenotypic features of CHH or reduced fertility [59].

### Robust candidate genes not currently included in the R148 panel “green list”

The following are examples of genes associated with CHH that have a strong body of evidence to support their candidacy as causal for the condition, and which might therefore be included in future development of the R148 panel “green” criteria gene list. Notably, several of these genes are already included in the NHS Scotland CHH gene panel (https://www.nhsggc.org.uk/media/271442/germlinetestdirectory_v10.pdf). Arguably, any of the following genes have equivalent, if not more, evidence behind them to genes such as *WDR11*.

### “Amber list” genes

#### *GNRH1* (MIM 152760)

*GNRH1* (Gonadotropin releasing hormone 1) is located on chromosome 8 and encodes the preprohormone that is processed to produce GnRH. Whilst this ligand is the prime candidate for the aetiology of GnRH deficiency conditions, the first study to pinpoint its role in the development of CHH was in 2009. A homozygous frameshift variant in *GNRH1* (NM_001083111.1: c.18–19insA, p.L18-19insA) was identified in a brother and sister with normosmic CHH. Both unaffected parents and unaffected sister were heterozygous carriers of this variant. Functional testing confirmed this to be a loss-of-function variant [60]. In the same year, screening of the *GNRH1* gene in 310 CHH patients identified a homozygous frameshift variant (NM_001083111.1: c.87delA, p.G29Gfs*12) in a patient with severe CHH. The variant was demonstrated to lead to the expression of a truncated protein [61]. Four further variants in *GNRH1* were subsequently identified in individuals with CHH, including a missense variant at a putative hot spot at arginine 31 (NM_0001083111.1: c.91C>T, p.R31C) found in 4 families [62]. This variant has been seen with homozygous carriage in CHH patients and with heterozygous carriage both in individuals with CHH and with isolated delayed puberty [62, 63]. Molecular characterisation of GNRH1 p.R31C mutant protein and reference GNRH decapeptides showed that the former had a 100-fold decrease in its affinity to bind its receptor, GnRH-R, compared to wild type GnRH. GNRH1 p.R31C mutant protein also showed reduced ability to activate the MAPK pathway and to trigger inositol phosphate accumulation and intracellular calcium mobilisation [62].

#### *FEZF1* (MIM 613301)

*FEZF1* (FEZ family zinc finger 1) encodes a transcriptional repressor, which is selectively expressed during embryogenesis. It is highly expressed in the amygdala, olfactory epithelium and hypothalamus, key sites for the margination of GnRH neuronal migration to the forebrain from the nasal placode [64]. Kotan et al screened 30 probands using a combination of autozygosity mapping and exome sequencing and identified two homozygous variants of interest (NM_001024613.2: c.832C>T, p.H278Y; c.652del, p.A217fs*13) in *FEZF1* in two independent consanguineous families with two affected siblings from each family [65]. The affected individuals had severe KS with absent olfactory bulbs, anosmia and complete hypogonadotropic hypogonadism, while unaffected heterozygous parents and siblings had normal olfactory and reproductive functions. Functional consequence of the mutant proteins was then assessed by measuring their transcriptional activity to supress Hes5 (hes family bHLH transcription factor 5). Mutant FEZF1 showed significant impairment of Hes5 downregulation [65]. In another study, whole exome sequencing of DNA samples from a cohort of seven KS probands from three independent families, with olfactory bulb dysplasia and delayed puberty, and Sanger sequencing of the patients and their relatives, identified novel trigenic variants in *PROKR2*, *CHD7* and *FEZF1* in one of the KS families [66]. Animal experiments demonstrated that *Fezf1*- deficient mice displayed a defect of olfactory receptor neuronal axonal projection into the basal lamina of the CNS. These mice lacked GnRH neurons in the brain, had smaller olfactory bulbs, and died shortly after birth [64].

### “Red list” genes

#### *SOX10* (MIM 602229)

*SOX10* (SRY-box 10) encodes a transcription factor that belongs to the SOX (SRY-related HMG-box) family of transcription factors, which are involved in modulating embryonic development and plays a key role in determining cell fate. Pathogenic variants in *SOX10* are associated with Waardenburg Syndrome, a rare group of disorders associated with sensorineural hearing loss and pigmentation deficiencies [67]. Pingault et al. reported that one-third of KS patients with deafness present with loss of function mutations in SOX10 [68]. Animal experimentation revealed that Sox10- deficient mouse exhibited an absence of subpopulation of glial cells called olfactory ensheathing cells along the olfactory nerve pathway, defective migration of GnRH cells and disorganisation of the olfactory nerve layer of the olfactory bulbs [68].

### Genes under evaluation for inclusion in the R148 panel

#### *NDNF* (MIM 616506)

*NDNF* (Neuron-derived neurotrophic factor) is a very recently identified potential causal gene in CHH, with pathogenic variants identified in four families in a sole publication [69]. The authors focused on variant finding in genes belonging to the fibronectin-3 (FN3) superfamily, as several genes encoding peptides that contain this conserved domain (*LEPR*, *AXL*, *FLRT3* and *DCC*) have been implicated in CHH or KS. As a result, the study identified three unrelated CHH probands with heterozygous protein truncating mutations in *NDNF* (NM_024574.3: c.184A>T, p.K62^∗^; c.381del, p.Y128Tfs^∗^55; c.1406G>A, p.W469^∗^) and an additional heterozygous missense variant (NM_024574.3: c.602C>G, p.T201S). All patients had severe GnRH deficiency and anosmia consistent with KS. One variant was also carried by a proband’s father, who had anosmia but no other features of KS, suggesting incomplete penetrance in this pedigree. Knockdown of NDNF affected the GnRH axis in zebrafish and mice models [69].

### Genes not yet on the R148 list

#### *SEMA3A* (MIM 603961)

*SEMA3A* (Semaphorin 3A) was first linked to the aetiology of Kallmann syndrome by the identification of a heterozygous 213-kb deletion in an individual with from a non-consanguineous family with several affected individuals. This deletion co-segregated with KS status in the family in an autosomal dominant pattern [70]. Furthermore, Hanchate et al (2012), screened all exons of the *SEMA3A* gene and its flanking regions in 386 KS patients with Sanger sequencing and identified several variants in 24 patients, five of which also presented with heterozygous variants of interest in other well-known KS associated genes (*PROKR2*, *FGFR1, PROK2*, and *KAL1*) [71]. The *SEMA3A* variants identified in KS patients included heterozygous missense variants (NM_006080: c.197C>T, p.R66W; c.458A>G, p.N153S; c.1198A>G, p.I400V; c.1303G>A, p.V435I; c.2062A>G, p.T688A; c.2189G>A, p.R730Q; c.2198G>A, p.R733H) and a heterozygous frameshifting small deletion (NM_006080: c.del1613_1626, p.D538fsX31). Functional analyses of these variants showed that seven mutations either showed defective secretion of semaphorin-3A, or decreased signalling activity of the secreted protein [71]. Animal studies in the same study involved creating a KS-like phenotype, Nrp1^sema/sema^ mutant mice, which lack the ability of Sema3a to bind to its obligatory coreceptor Nrp1, and showed abnormal GnRH cell migration into the basal forebrain [71].

Further studies in a Finnish CHH patient cohort [72], identified heterozygous variants in the SEMA3A gene in three KS patients (NM_006080.2: c.458A>G, p.N153S; c.1253A>G, p.N418S; c.1303G>A, p.V435I), two of which had a previously reported mutation in the *FGFR1* gene. Studies in Chinese cohorts identified novel loss-of-function *SEMA3A* variants (NM_006080: c.1369A>G, p.T457A; c.1850G>A, R197Q; c.1850G>A, R617Q; c.1372G>A, V458I) in male normosmic CHH patients, some of whom also carried variants in other genes including *PLXNA4*, *PLXND1* and *FGFR1* [73, 74]. The authors suggested that *SEMA3A* variants might have a role in modifying the CHH phenotype.

## Discussion

The development and clinical availability of a high throughput sequencing panel represents a major step forward to facilitate molecular genetic diagnosis in patients with CHH. As for all genetic diagnostic testing, there is a balance to be struck between identifying a definitively pathogenic and clinically actionable variant in a small number of highly curated causal genes, and inclusivity of a larger number of genes that have evidence of association with a disease. The implementation of a small, focused panel such as the R148 gene panel allows the potential for genetic diagnosis in the clinic, which was previously only available in a research setting. However, its utility is dependent on the clinician’s understanding that there are a limited number of candidate genes included in the panel and that a ‘negative’ result does not exclude the diagnosis of CHH. Moreover, the exclusion of multiple genes strongly implicated in the aetiology of the condition, such as *GNRH1*, from the panel “green” list may limit the potential to make a genetic diagnosis in a timely manner. Accurate diagnosis, particularly in adolescence, is vital to facilitate early intervention in these patients to assist with the management of associated health and psychosocial sequelae such as low bone density, fractures, subfertility and emotional distress.

Alternatively, sequencing of these patients by whole exome sequencing would allow exploration of both the large pool of alternative CHH candidates genes and novel gene discovery. Further, it would expand the potential to identify di- or oligogenic carriage in an individual who might otherwise be diagnosed with a monogenic inheritance. A very recent survey of the current practice in European centres shows that while the majority of centres are using candidate panel analysis for CHH diagnostics, a small number of centres already offer whole exome sequencing analysis at initial testing, with a clinical report given on a virtual candidate gene panel [75]. The informative mutation yields are, as expected, much higher with such an approach. Custom or virtual panels reporting large numbers of genes are more likely to report an identified pathogenic variant in up to 60% of cases, whereas positive results fall below 15% in centres using small panels such as the R148 panel.

In the future, an optimal pathway might include patients sent for clinical panel testing having whole exome (or genome) sequencing, with secondary implementation of the carefully defined gene panel “virtually” as part of the bioinformatics filtering pipeline. This would facilitate frequent review of the curation of the “green” list genes (that are clinically reportable) within the panel by senior clinical geneticists in conjunction with expert clinicians. This is particularly relevant for a condition such as CHH for which, as illustrated above, a high number of new publications on novel and existing potentially causal genes are published each year. It would also allow the development of ‘sub-panels’ within a condition, for example a syndromic CHH/KS panel for an individual with relevant associated phenotypes.

## Conclusion

The implementation of a CHH panel in clinical endocrine diagnostics is a positive development to facilitate optimal patient care, as well as to provide a foundation for genetic counselling, fertility treatment and family planning. However, clinicians need to be aware of the practical considerations implicit in designing such a panel and its limitations. Future avenues may include the use of whole exome or genome sequencing with frequently updated virtual panels to filter clinically actionable results.
